# Increased risk of cervical dysplasia in females with autoimmune conditions—Results from an Australia database linkage study

**DOI:** 10.1371/journal.pone.0234813

**Published:** 2020-06-18

**Authors:** Emma Foster, Michael J. Malloy, Vilija G. Jokubaitis, C. David H. Wrede, Helmut Butzkueven, Joe Sasadeusz, Sharon Van Doornum, Finlay Macrae, Gary Unglik, Julia M. L. Brotherton, Anneke van der Walt

**Affiliations:** 1 Department of Neurology, MS and Neuroimmunology Service, Alfred Health, Melbourne, Australia; 2 Department of Neuroscience, Central Clinical School, Monash University, Melbourne, Australia; 3 Department of Neurology, Royal Melbourne Hospital, Melbourne, Australia; 4 Victorian Cervical Screening Registry, VCS Population Health, VCS Foundation, Melbourne, Australia; 5 Melbourne School of Population and Global Health, University of Melbourne, Melbourne, Australia; 6 Department of Oncology and Dysplasia, Royal Women’s Hospital, Melbourne, Australia; 7 Department of Obstetrics and Gynaecology, The University of Melbourne, Melbourne, Australia; 8 Victorian Infectious Diseases Service, The Royal Melbourne Hospital, Melbourne, Australia; 9 Rheumatology Department, The Royal Melbourne Hospital, Melbourne, Australia; 10 Department of Medicine, The University of Melbourne, Melbourne, Australia; 11 Colorectal Medicine and Genetics, The Royal Melbourne Hospital, Melbourne, Australia; 12 Department of Clinical Immunology and Allergy, The Royal Melbourne Hospital, Melbourne, Australia; Rudjer Boskovic Institute, CROATIA

## Abstract

**Background:**

Autoimmune conditions (AICs) and/or their treatment may alter risk of human papilloma virus (HPV) infection and females with AICs are therefore at an increased risk of cervical dysplasia. However, inclusion of these at-risk populations in cervical cancer screening and HPV-vaccination guidelines, are mostly lacking. This study aimed to determine the prevalence of cervical dysplasia in a wide range of AICs and compare that to HIV and immunocompetent controls to support the optimisation of cervical cancer preventive health measures.

**Methods:**

Data linkage was used to match cervical screening episodes to emergency department records of females with AICs or HIV to immunocompetent controls over a 14-year period. The primary outcome was histologically confirmed high-grade cervical disease. Results, measured as rates by cytology and histology classification per 1,000 females screened, were analysed per disease group, and intergroup comparisons were performed.

**Results:**

Females with inflammatory bowel disease (2,683), psoriatic and enteropathic arthropathies (1,848), multiple sclerosis (MS) (1,426), rheumatoid arthritis (1,246), systemic lupus erythematosus and/or mixed connective tissue disease (SLE/MCTD) (702), HIV (44), and 985,383 immunocompetent controls were included. SLE/MCTD and HIV groups had greater rates of high-grade histological and cytological abnormalities compared to controls. Increased rates of low-grade cytological abnormalities were detected in all females with AICs, with the exception of the MS group.

**Conclusions:**

Females with SLE/MCTD or HIV have increased rates of high-grade cervical abnormalities. The increased low-grade dysplasia rate seen in most females with AICs is consistent with increased HPV infection. These findings support expansion of cervical cancer preventative programs to include these at-risk females.

## Introduction

Human papillomavirus (HPV) is the most common sexually transmitted infection, and high-risk types such as HPV 16 and 18 are oncogenic and associated with pre-malignant and malignant conditions of the cervix and anogenital lesions [1]. Control of HPV infection relies upon an effective local immune response and therefore diseases associated with impaired immunity, either due to the disease itself or immunomodulatory treatment, can increase the risk of HPV-related conditions [2]. Heavily immunocompromised females, such as those with untreated human immunodeficiency virus (HIV), are most at risk of cervical, vaginal or vulval intra-epithelial dysplasia and HPV-related cancers [3,4]. Population-wide cohort studies have shown an increased risk of cervical abnormalities in females with autoimmune conditions including inflammatory bowel disease (IBD) [5], systematic lupus erythematosus (SLE) [6], and rheumatoid arthritis (RA) [7], especially if treated with immunomodulatory therapy. There is limited data regarding the risk of persistent HPV infection, cervical dysplasia and HPV-related cancers in females with multiple sclerosis (MS) [8].

Despite the existing evidence, cervical cancer prevention guidelines typically lack a comprehensive approach to these at-risk individuals even though many countries do recommend more frequent cervical screening in immunocompromised individuals [9]. Prophylactic HPV vaccination is a complementary public health measure to cervical screening. However, the optimal use of the vaccine in immunocompromised populations is not well defined [9]. National guidelines in many countries recommend a two-dose schedule for immunocompetent females aged 11–14 years, as the vaccine is most immunogenic at a younger age and confers the greatest benefit when given prior to HPV exposure (i.e. before first sexual activity) [10]. Three doses are recommended in those immunocompromised at time of vaccination (regardless of age) and for those aged 15 years or older at first dose.

Australia is a world leader in cervical cancer prevention and is predicted to be one of the first countries to achieve elimination of cervical cancer as a public health problem [11]. However, the lack of local data on the burden of disease amongst immunocompromised females has arguably resulted in delays optimising public health policy. Autoimmune conditions have a preponderance in young to middle aged females and many would not have had the opportunity to receive the HPV vaccine as teenagers. In addition, the financial barrier posed by recommending HPV vaccination outside of the targeted age range is substantial, and, data supporting the potential benefits of an expanded indication for prophylactic vaccination in this group are required.

Here, we report and compare rates of cervical abnormalities in females with a wide range of autoimmune conditions in Australia. We included SLE/mixed connective tissue disease (MCTD), RA, psoriatic and enteropathic arthropathies (PsA and EA), IBD, MS and primary immunodeficiencies or HIV and comparison with an immunocompetent community cohort.

## Methods

We undertook a retrospective cohort study utilising data collected from 1 January 2000 to 31 December 2013 in the state of Victoria, Australia. In 2006, the mid-point of the study period, Victoria’s population was 5.13 million with 50.5% female [12]. Inclusion criteria were females aged 15 and over who attended any public Victorian hospital emergency department, as identified through the Victorian Emergency Minimum Dataset (VEMD) and had ever undergone community cervical screening in the study period, as identified through deterministic data linkage to the Victorian Cervical Cytology Registry (VCCR). The VCCR recorded cervical screening cytology and histopathology results for Victorian females, with < 1% opting off the registry [13]. Females identified during the study period to have an autoimmune condition, primary immunodeficiency or HIV as per VEMD discharge diagnoses (S1 Appendix) were assigned to that study group (S2 Appendix) for the duration of the study period. These study groups were compared to presumed immunocompetent controls (i.e. those females who did not have any episodes with these conditions identified via the VEMD). We excluded females recorded as having cervical cancer, hysterectomy or as deceased prior to entry into the study.

Deterministic matching was performed by the Centre for Victorian Data Linkage (CVDL) to identify females common to both VCCR and VEMD. Analytic variables used for matching were name, address, postcode, Medicare number, sex and date of birth. VCCR variables returned to investigators for analysis were age (month/year of birth), cervical screening history at the episode level and postcode (used to assign remoteness area and area level socioeconomic status). VEMD variables were age (month/year of birth), Aboriginal and Torres Strait Islander status, country of birth, reason for attendance (coded according to ICD10-AM) and attendance date (as month/year), and statistical local area.

Histological and cytological outcomes were assigned according to Australian Institute of Health and Welfare (AIHW) categorisation and Australian Standardised Modified Bethesda System, respectively [14]. The primary outcome was histologically confirmed high-grade cervical disease defined as cervical intra-epithelial neoplasia (CIN2, CIN3, adenocarcinoma in situ (AIS) or mixed CIN3/AIS). A female that had multiple outcomes in the study period, e.g. both a cytological low-grade, and a histological high-grade abnormality, was counted for each outcome independently in separate analyses run for each outcome. Females needed to have two negative tests over two years in order for a second abnormality to be recorded as an incident abnormality; two negative Pap tests was assumed to equate to a confirmed clearance period. Person-time was counted until the outcome of interest, date of death, hysterectomy, 2.5 years post their last negative cytology test, or the end of the study period, whichever came first.

### Ethics approval and consent

This study was approved by the Victorian Department of Health and Human Services Human Research Ethics Committee (study number 01/11). All data were fully anonymized before researchers accessed them, and ethics committee waived the requirement for informed consent.

### Statistical methods

The Mann-Whitney U test was applied to compare the means of the continuous variables and Pearson chi-squared test for nominal variables. Where a comparison contained expected frequencies < 10, Fisher’s exact test was applied. Normality of continuous variables was assessed using the Shapiro-Wilk test. Cox regression, with age as the time axis, was used to estimate hazard ratios (HR) with 95% confidence intervals (CI) of cervical abnormalities according to study group status. Using an entry-age-adjusted age-scale as the time axis allows the baseline hazard to change as a function of age, better controlling for potential confounding due to age [15]. Covariates analysed included number of screening episodes, area level socioeconomic status assigned from postcode in quintiles using the Australian Bureau of Statistics Socio-Economic Indexes for Areas Index of Relative Socio-Economic Disadvantage [16], remoteness area assigned from postcode using the Australian Standard Geographic Classification for 2011 [17], Aboriginal and/or Torres Strait Islander status, and country of birth in Major Categories (Appendix 3) [18]. Any covariates that violated the Cox model assumption of proportional hazards were stratified out of each model. Kaplan-Meier curves were derived using time-on-study, i.e. follow-up time, as the time axis. Incidence rates by cytology and histology classification were calculated per disease group and on the scale of per 1,000 person-years. Intergroup comparisons were then performed.

## Results

**Fig 1:**
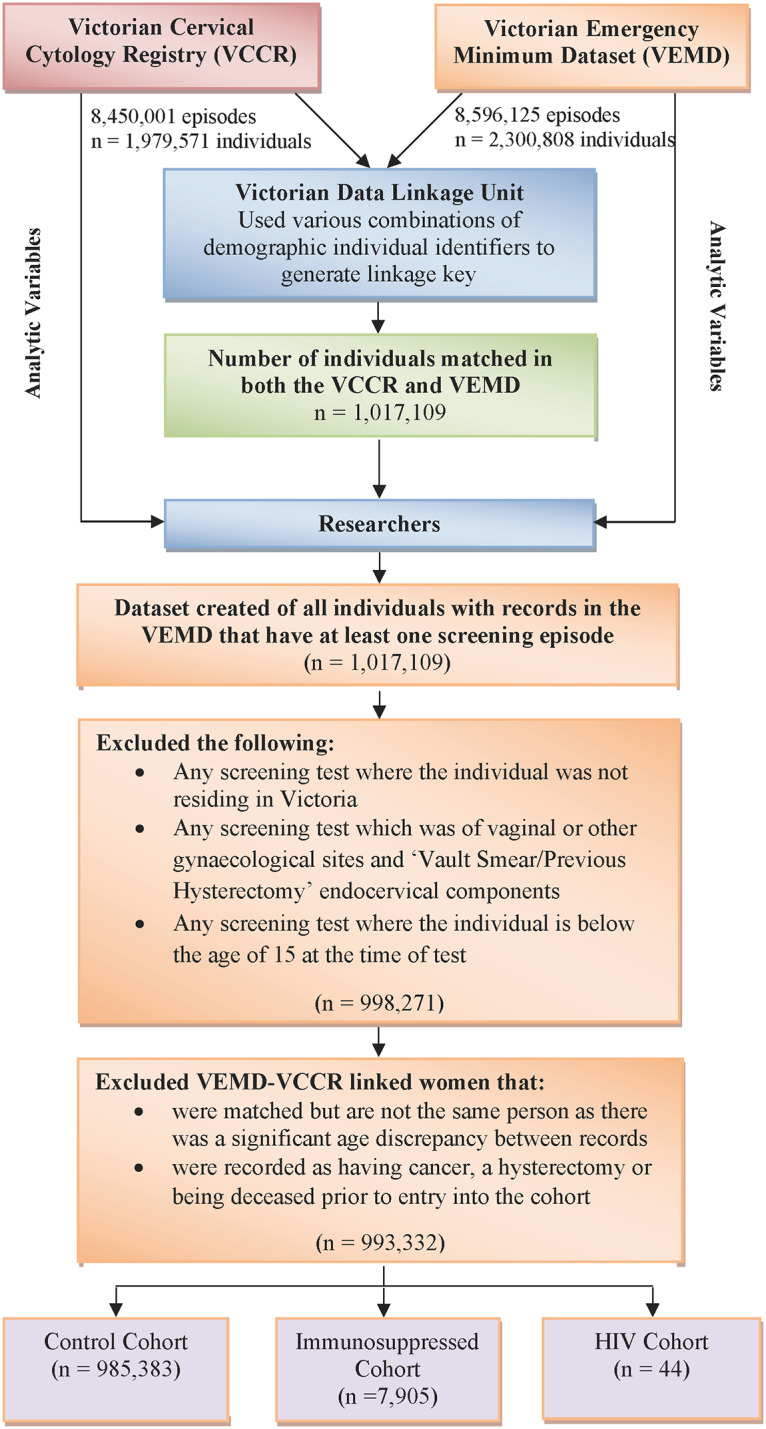
Flowchart of data linkage process and exclusions for analysis.

We identified 985,383 females in the control cohort, 7,905 in the autoimmune condition cohort, 44 in the HIV cohort and none in the primary immunodeficiency cohort over the 14-year study period (Fig 1). The overall median follow-up time from study entry to end-point was 9.43 years. Of the females with an autoimmune condition, 2,683 (33.9%) were diagnosed with IBD, 1,848 (23.4%) PsA and EA, 1,426 (18.0%) with MS, 1,246 (15.8%) with RA and 702 (8.9%) with SLE/MCTD.

Compared to controls, females in the autoimmune condition cohort had higher mean age at study entry (35.5 years vs 34.8 years, p<0.001), were less likely to live in major cities (71.3% vs 75.7%, p<0.001), were more likely to be in the lowest socioeconomic bracket (21.9% vs 18.3%, p<0.0001) and to identify as Aboriginal or Torres Strait Islander (1.5% vs 1.1%, p = 0.001). (Table 1) On average, the mean number of Pap tests performed was significantly higher for females in the autoimmune cohort (n = 4.48, p<0.001) and HIV cohort (n = 5.64, p<0.035) compared to controls (n = 4.35). Females in the autoimmune condition cohort with abnormalities detected had a significantly higher mean number of Pap tests compared to controls with abnormalities (n = 6.57 vs n = 6.35, p = 0.003).

**Table 1:** Descriptive characteristics of the control, auto-immune condition (AIC) and HIV cohorts.

	Control	AIC	P-value^	HIV	P-value^#^
**Number of individuals** ^a^	985,383	7,905		44	
**Mean age at entry to study in Years (SD)**	34.79 (±13.16)	35.52 (±13.09)	<0.001	33.64 (±11.02)	0.840
**Mean number of Pap tests (SD)**	4.35 (±2.79)	4.48 (±2.87)	<0.001	5.64 (±4.01)	0.035
**Mean number of Pap tests by abnormality status (SD)**					
No abnormalities	3.75 (±2.35)	3.76 (±2.33)	0.696	4.05 (±2.24)	0.460
One or more abnormalities	6.35 (±3.20)	6.57 (±3.23)	0.003	7.23 (±4.75)	0.647
**Age at entry to study (Years)**					
<30	421,587 (42.78%)	3,132 (39.62%)	<0.001	18 (40.91%)	0.802
30–39	240,747 (24.43%)	2,053 (25.97%)	0.002	12 (27.27%)	0.661
40–49	161,484 (16.39%)	1,397 (17.67%)	0.002	9 (20.45%)	0.420*
50–59	106,424 (10.80%)	845 (10.69%)	0.752	5 (11.36%)	0.809*
> = 60	55,141 (5.60%)	478 (6.05%)	0.082	0 (0%)	0.177*
**Remoteness area** ^b^					
Major cities	743,978 (75.67%)	5,619 (71.26%)	<0.001	32 (74.42%)	0.848
Inner regional	197,510 (20.09%)	1,886 (23.92%)	<0.001	8 (18.60%)	1.000*
Outer regional	41,376 (4.21%)	376 (4.77%)	0.014	3 (6.98%)	0.428
Remote/Very Remote	317 (0.03%)	4 (0.05%)	0.331*	0 (0%)	1.000*
**Socioeconomic status** ^c^					
1 (Lowest)	179,394 (18.30%)	1,725 (21.92%)	<0.001	10 (23.26%)	0.428*
2	138,478 (14.12%)	1,251 (15.90%)	<0.001	4 (9.30%)	0.510*
3	170,748 (17.42%)	1,442 (18.33%)	0.034	8 (18.60%)	0.840*
4	227,866 (23.24%)	1,822 (23.16%)	0.859	7 (16.28%)	0.280
5 (Highest)	263,910 (26.92%)	1,628 (20.69%)	<0.001	14 (32.56%)	0.404
**Cytological abnormalities diagnosed on entry into cohort** ^d^					
Cancer	80 (0.01%)	1 (0.01%)	0.477*	0 (0%)	1.000*
High-grade					
Definite	8,459 (0.86%)	75 (0.95%)	0.389	4 (9.09%)	0.001*
Possible	5,913 (0.60%)	45 (0.57%)	0.721	1 (2.27%)	0.233*
Glandular	85 (0.01%)	1 (0.01%)	0.497*	0 (0%)	1.000*
Low-grade	54,065 (5.49%)	488 (6.17%)	0.008	6 (13.64%)	0.032*
Negative	897,539 (91.14%)	7,151 (90.46%)	0.034	32 (72.73%)	<0.001*
Unsatisfactory	18,646 (1.89%)	144 (1.82%)	0.641	1 (2.27%)	0.569*
**Aboriginal & Torres Strait Islander Status** ^e^					
Aboriginal Only	6,484 (0.66%)	95 (1.20%)	<0.001	0 (0%)	1.000*
Torres Strait Islander Only	402 (0.04%)	3 (0.04%)	1.000*	0 (0%)	1.000*
Aboriginal & Torres Strait Islander	1,751 (0.18%)	15 (0.19%)	0.800	1 (2.27%)	0.075*
Neither Aboriginal or Torres Strait Islander	974,541 (98.90%)	7,788 (98.52%)	0.001	43 (97.73%)	0.385*
Question unable to be asked	1,843 (0.19%)	4 (0.05%)	0.005	0 (0%)	1.000*
Patient refused to answer	362 (0.04%)	0 (0%)	0.127*	0 (0%)	1.000*
**Country of Birth (Major Categories)** ^f^					
Americas	12,724 (1.29%)	89 (1.13%)	0.194	0 (0%)	1.000*
North Africa & The Middle East	22,826 (2.32%)	206 (2.61%)	0.088	1 (2.27%)	1.000*
North-East Asia	19,859 (2.02%)	56 (0.71%)	<0.001	1 (2.27%)	0.592*
North-West Europe	54,222 (5.50%)	457 (5.78%)	0.280	1 (2.27%)	0.517*
Oceania & Antarctica	730,329 (74.12%)	6,186 (78.25%)	<0.001	32 (72.73%)	0.833
South-East Asia	32,519 (3.30%)	141 (1.78%)	<0.001	4 (9.09%)	0.057*
Southern & Central Asia	27,079 (2.75%)	125 (1.58%)	<0.001	1 (2.27%)	1.000*
Southern & Eastern Europe	60,659 (6.16%)	524 (6.63%)	0.082	1 (2.27%)	0.523*
Sub-Saharan Africa	12,069 (1.22%)	91 (1.15%)	0.553	3 (6.82%)	0.017*
Not Classified	13,097 (1.33%)	30 (0.38%)	<0.001	0 (0%)	1.000*

SD = Standard deviation, AIC = Autoimmune Condition, HIV = Human Immunodeficiency Virus

^ P-value compares cases against controls

^#^ P-value compares HIV cohort against controls

* P-value calculated using Fisher’s Exact Chi-Squared test.

^a^ The total number of individuals is 993,332.

^b^ Individuals were allocated to a remoteness area based on their postcode of usual residence, according to the Australian Standard Geographic Standard (ASGS) for 2011. Missing data on 2,223 individuals (0.22%; 2,202 controls, 20 cases: Crohn’s x 2, MS x 2, Arthropathies x 6, RA x 2, SLE x 1, UC x 7 and 1 HIV) were excluded.

^c^ Individuals were allocated to a socioeconomic status (SES) groups based on their postcode of usual residence, according to the Australian Bureau of Statistics Socio-Economic Indexes for Areas (SEIFA) Index of Relative Socio-Economic Disadvantage. Missing data on 5,025 individuals (0.51%; 4,987 controls, 37 cases: Crohn’s x 7, MCTD x 1, MS x 3, Arthropathies x 11, RA x 4, SLE x 2, UC x 9 and 1 HIV) were excluded.

^d^ 596 individuals (0.06%; 596 controls) were excluded as they only had histological episodes.

^e^ Aboriginal & Torres Strait Islander classification was determined by using the ‘Ever Aboriginal & Torres Strait Islander’ method

^f^ Country of Birth classification was determined by using the ‘Majority’ method

### Outcome data and main results

**Fig 2:**
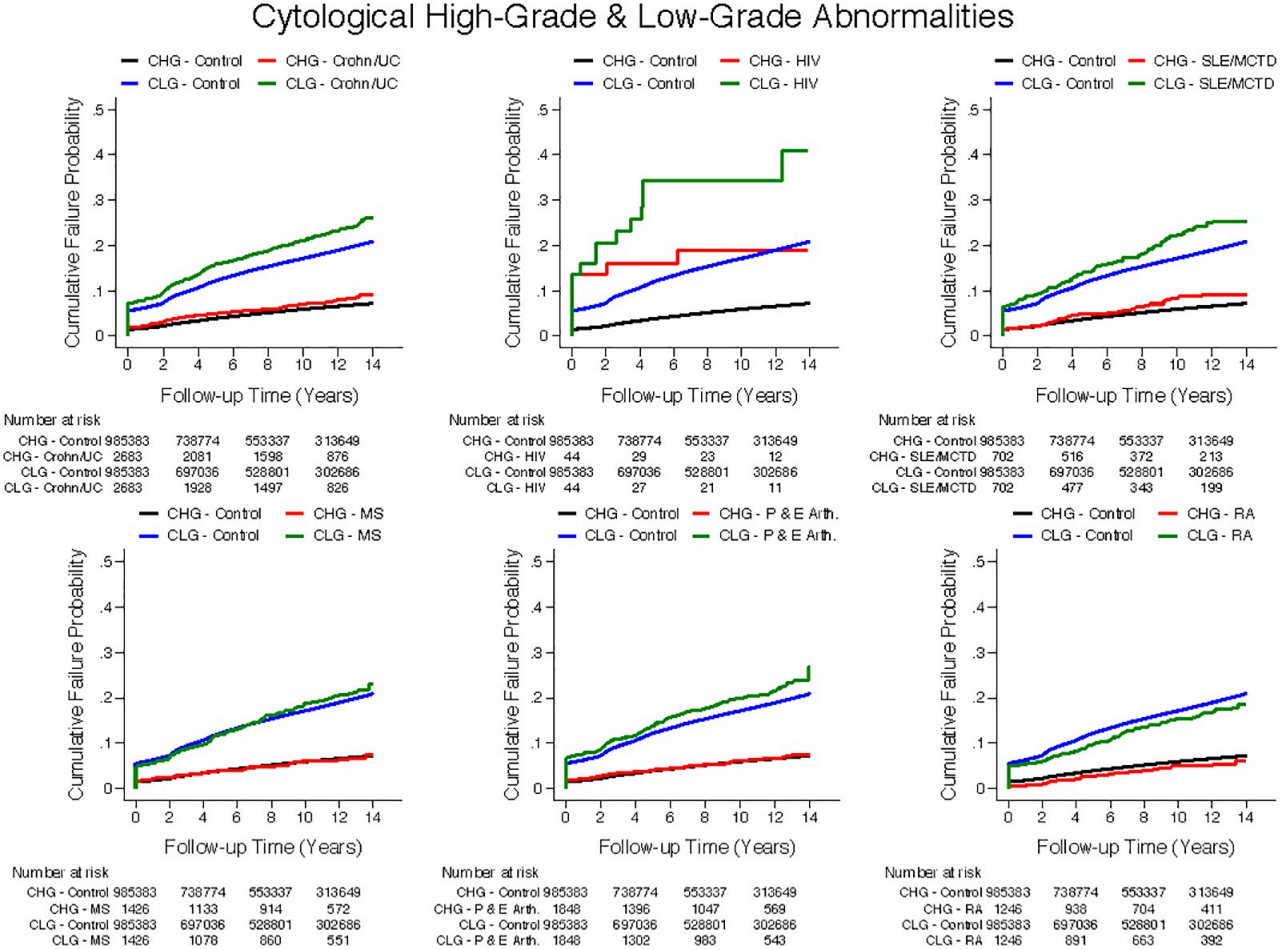
Cumulative incidence of cytological high-grade and low-grade abnormalities. Cumulative incidence of cytological high grade (CHG) and Cytological low grade (CLG) abnormalities compared to healthy controls in a) Crohn’s disease/Ulcerative colitis. b) HIV c) SLE/MCTD d) MS e) PsA and EA and f) RA. UC = Ulcerative colitis; HIV = Human immunodeficiency virus; SLE/MCTD = Systemic Lupus Erythematosus/ Mixed Connective Tissue Disease; MS = multiple sclerosis; P&E = psoriatic arthritis and enteropathic arthropathy; RA = Rheumatoid arthritis.

**Fig 3:**
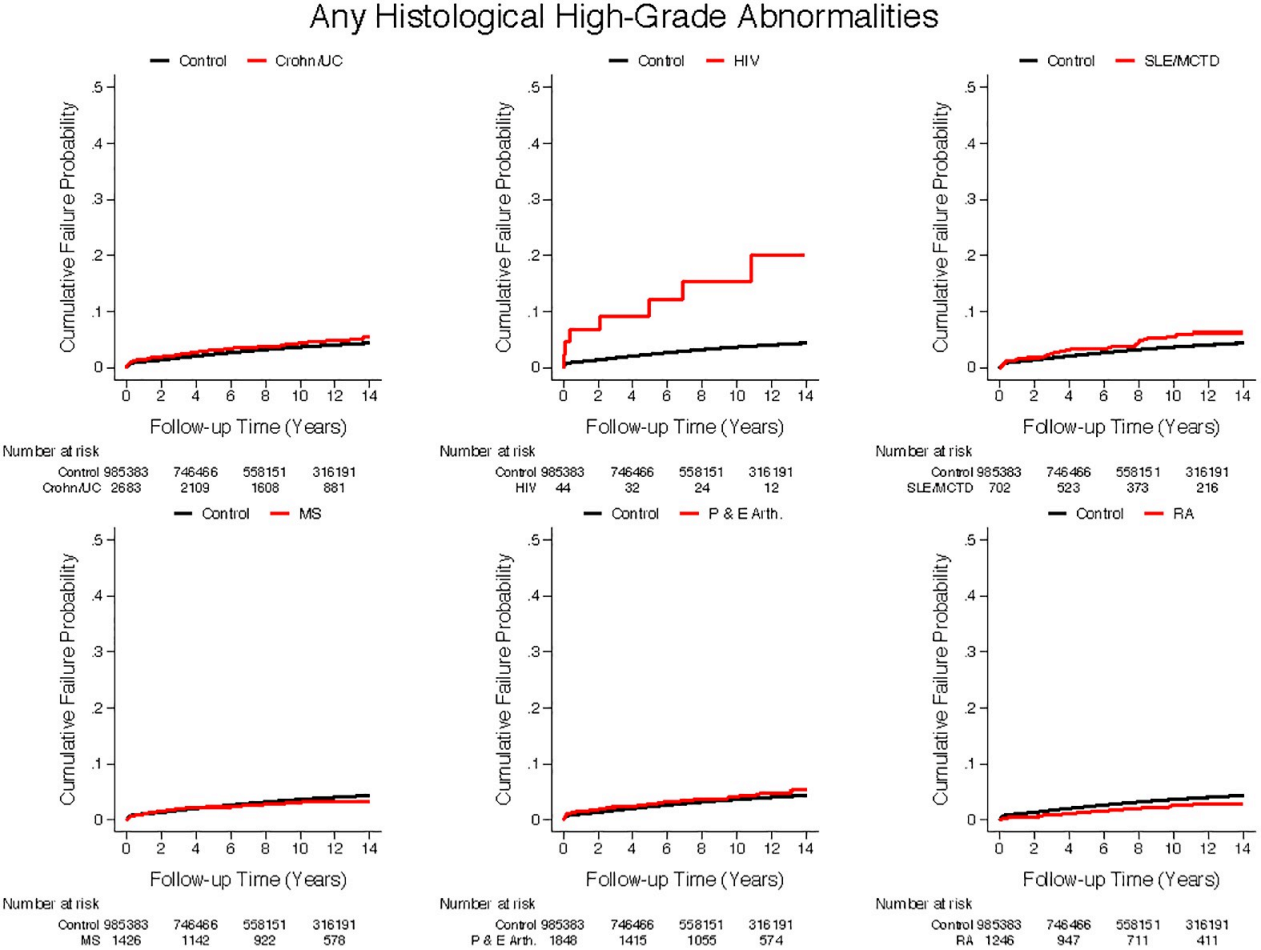
Cumulative incidence of histological high-grade abnormalities. Cumulative incidence of histological high grade abnormalities compared to healthy controls in a) Crohn’s disease/Ulcerative colitis. b) HIV c) SLE/MCTD d) MS e) Psoriatic and enteropathic arthropathy and f) RA. UC = Ulcerative colitis; HIV = Human immunodeficiency virus; SLE/MCTD = Systemic Lupus Erythematosus/ Mixed Connective Tissue Disease; MS = multiple sclerosis; P&E = psoriatic arthritis enteropathic arthropathy; RA = Rheumatoid arthritis.

SLE/MCTD and HIV groups had significantly higher rates of high-grade histological and cytological abnormalities than controls. These groups, along with IBD, PsA, EA and RA groups, also had significantly higher rates of low-grade cytological abnormalities than controls (Table 2). Figs 2 and 3 display cumulative incidence of low- and high-grade cytological abnormalities and of high-grade histological abnormalities for conditions of interest compared to controls.

**Table 2:** Number, incidence rate and adjusted hazard ratios for cervical abnormalities of the control, auto-immune conditions and HIV cohorts.

Outcome	Cohort	Number of Women	Number of Abnormalities	Person-Years	Rate*	95% CI	AHR	95% CI	P-value
**Histological abnormalities**									
All high-grade histology^a^	Control	985,383	30,963	8,241,438.70	3.76	3.72 to 3.80	Reference		
	AIC	7,905	274	68,046.96	4.03	3.58 to 4.53	1.03	0.91 to 1.16	0.654
	HIV	44	7	360.83	19.40	9.25 to 40.69	4.89	2.33 to 10.27	<0.001
	*IBD*	*2*,*683*	*107*	*23*,*250*.*72*	*4*.*60*	*3*.*81 to 5*.*56*	*1*.*02*	*0*.*85 to 1*.*24*	*0*.*824*
	*SLE*, *MCTD*	*702*	*32*	*5*,*746*.*52*	*5*.*57*	*3*.*94 to 7*.*87*	*1*.*47*	*1*.*03 to 2*.*09*	*0*.*033*
	*MS*	*1*,*426*	*40*	*13*,*021*.*47*	*3*.*07*	*2*.*25 to 4*.*19*	*0*.*78*	*0*.*57 to 1*.*07*	*0*.*124*
	*PsA and EA*	*1*,*848*	*70*	*15*,*559*.*44*	*4*.*50*	*3*.*56 to 5*.*69*	*1*.*11*	*0*.*88 to 1*.*41*	*0*.*367*
	*RA*	*1*,*246*	*25*	*10*,*468*.*81*	*2*.*39*	*1*.*61 to 3*.*53*	*0*.*96*	*0*.*65 to 1*.*43*	*0*.*856*
**Cytological abnormalities**									
High-grade cytology^a^	Control	985,383	51,143	8,168,425.90	6.26	6.21 to 6.32	Reference		
	AIC	7,905	451	67,415.89	6.69	6.10 to 7.34	1.06	0.97 to 1.17	0.196
	HIV	44	8	335.59	23.84	11.92 to 47.67	3.43	1.64 to 7.21	0.001
	*IBD*	*2*,*683*	*177*	*23*,*036*.*70*	*7*.*68*	*6*.*63 to 8*.*90*	*1*.*12*	*0*.*96 to 1*.*29*	*0*.*144*
	*SLE*, *MCTD*	*702*	*47*	*5*,*697*.*80*	*8*.*25*	*6*.*20 to 10*.*98*	*1*.*33*	*1*.*00 to 1*.*78*	*0*.*051*
	*MS*	*1*,*426*	*77*	*12*,*882*.*54*	*5*.*98*	*4*.*78 to 7*.*47*	*0*.*98*	*0*.*78 to 1*.*22*	*0*.*836*
	*P & E Arthropathies*	*1*,*848*	*101*	*15*,*404*.*55*	*6*.*56*	*5*.*39 to 7*.*97*	*0*.*97*	*0*.*80 to 1*.*18*	*0*.*769*
	*Rheumatoid Arthritis*	*1*,*246*	*49*	*10*,*394*.*30*	*4*.*71*	*3*.*56 to 6*.*24*	*1*.*03*	*0*.*78 to 1*.*37*	*0*.*820*
Low-grade cytology^b^	Control	985,383	155,415	7,773,743.60	19.99	19.89 to 20.09	Reference		
	AIC	7,905	1,444	63,353.00	22.79	21.65 to 24.00	1.17	1.11 to 1.24	<0.001
	HIV	44	15	309.50	48.46	29.22 to 80.39	2.23	1.32 to 3.76	0.003
	*IBD*	*2*,*683*	*545*	*21*,*539*.*72*	*25*.*30*	*23*.*26 to 27*.*52*	*1*.*19*	*1*.*09 to 1*.*29*	*<0*.*001*
	*SLE*, *MCTD*	*702*	*137*	*5*,*263*.*87*	*26*.*03*	*22*.*01 to 30*.*77*	*1*.*36*	*1*.*15 to 1*.*61*	*<0*.*001*
	*MS*	*1*,*426*	*251*	*12*,*276*.*65*	*20*.*45*	*18*.*07 to 23*.*14*	*1*.*10*	*0*.*97 to 1*.*24*	*0*.*139*
	*P & E Arthropathies*	*1*,*848*	*342*	*14*,*412*.*69*	*23*.*73*	*21*.*34 to 26*.*38*	*1*.*12*	*1*.*01 to 1*.*25*	*0*.*035*
	*Rheumatoid Arthritis*	*1*,*246*	*169*	*9*,*860*.*06*	*17*.*14*	*14*.*74 to 19*.*93*	*1*.*23*	*1*.*05 to 1*.*42*	*0*.*008*

CI = Confidence Interval, AHR = Adjusted Hazard Ratio, AIC = Autoimmune Condition, HIV = Human Immunodeficiency Virus, IBD = Inflammatory Bowel Disease (Crohn’s Disease & Ulcerative Colitis), SLE = Systemic Lupus Erythematosus, MCTD = Mixed Connective Tissue Disease, MS = Multiple Sclerosis, P & E Arthropathies = Psoriatic & Enteropathic Arthropathies

* Rate per 1,000 person-years.

^a^ Cox model stratified by remoteness, country of birth and number of screening tests. Other co-variables adjusted for include: socio-economic status and indigenous status.

^b^ Cox model stratified by socio-economic status, country of birth and number of screening tests. Other co-variables adjusted for include: indigenous status.

All high grade histology defined as CIN2, CIN3, AIS and mixed CIN3/AIS.

High-grade cytology defined as possible high-grade squamous intraepithelial lesion (HSIL), HSIL, HSIL with possible microinvasion/invasion, squamous cell carcinoma, possible high-grade endocervical glandular lesion and AIS.

Low-grade cytology defined as possible low-grade squamous intraepithelial lesions (LSIL), LSIL and atypical endocervical cells of uncertain significance.

### HIV

Females with HIV had significantly higher rates of high-grade histological abnormalities (19.40 vs 3.76 per 1,000 person-years, AHR = 4.89, p<0.001), high-grade cytological abnormalities (23.84 vs 6.26 per 1,000 person-years, AHR = 3.43, p = 0.001) and low-grade cytological abnormalities (48.46 vs 19.99 per 1,000 person-years, AHR = 2.23, p = 0.003).

### SLE/MCTD

Females with SLE/MCTD had significantly higher rates of high-grade histological abnormalities (5.57 vs 3.76 per 1,000 person-years, AHR = 1.47, p = 0.033), significantly higher rates of high-grade cytological abnormalities (8.25 vs 6.26 per 1,000 person-years, AHR = 1.33, p = 0.051), and significantly higher rates of low-grade cytological abnormalities (26.03 vs 19.99 per 1,000 person-years, AHR = 1.36, p<0.001).

### IBD

Females with IBD had significantly higher rates of low-grade cytological abnormalities compared to controls (25.30 vs 19.99 per 1,000-person years, AHR = 1.19, p<0.001).

#### Psoriatic and enteropathic arthropathies

Females with PsA or EA had significantly higher rates of low-grade cytological abnormalities compared to controls (23.73 vs 19.99, AHR = 1.12, p = 0.035).

### RA

Females with RA had significantly higher AHR of low-grade cytological abnormalities compared to controls (17.14 vs 19.99 per 1,000 person-years, AHR = 1.23, p = 0.008).

### MS

Females with MS had no significant differences in rates of high-grade histological abnormalities (3.07 vs 3.76 per 1,000 person-years, AHR = 0.78, p = 0.124), high-grade cytological abnormalities (5.98 vs 6.26 per 1,000 person-years, AHR = 0.98, p = 0.836) or low-grade cytological abnormalities (20.45 vs 19.99 per 1,000 person-years, AHR = 1.10, p = 0.139) compared to controls.

## Discussion

This study suggests that females with HIV and with SLE/MCTD are at 5 times and 1.5 times greater risk, respectively, of high-grade cervical abnormalities compared to controls after adjustment for age-at-entry, socioeconomic status and screening frequency. These conditions, along with IBD, PsA, EA and RA, were also associated with greater rates of low-grade cytological abnormalities, indicating higher HPV infection rates. Similar to previously reported findings [8], we found no significant differences in rates of cervical abnormalities between females with MS and controls.

The increased incidence of cervical abnormalities in females with these conditions is consistent with a previous meta-analyses that showed an increased risk of cervical dysplasia in SLE (OR 4.17 (95% CI 3.03, 5.74) [3,6] and IBD (OR 1.34 (95% CI 1.23–1.46) compared to controls [5]. Similarly, Swedish registry data reported an increased risk low-grade cytological lesions in RA [7], while cervical dysplasia rates in PsA and EA have not been previously been reported.

Altered immune surveillance due to both intrinsic disease-related factors and/or immunomodulatory therapies have been postulated to contribute to this risk. Immunodeficiency and immune dysregulation frequently coexist in autoimmune disease. As such, patients with autoimmune conditions such as SLE, RA and psoriasis may present with disturbances in their immune system, and, conversely, primary immunodeficiencies can first present as autoimmune conditions [19]. MS is a possible exception to this given the single-tissue target, i.e. the central nervous system, of autoimmunity. In addition, the immune-pathogenesis of MS is increasingly recognized to evolve over the disease course from an initial peripheral, T-cell driven, immune response to a CNS dominant process later in the disease that is largely driven by the innate immune system [20].

Immune clearance and control of HPV requires cell mediated immunity and T-cell functioning, hence the observation of higher rates of infection and HPV-related diseases in conditions where such immune control is compromised [2]. A wider diversity of HPV types and multiple types of infections are more frequently detected amongst immunocompromised females. However, cervical dysplasia and cancer are still predominantly caused by the most oncogenic types, HPV 16 and 18, although to a lesser extent than amongst immunocompetent females. The low risk types HPV 6 and 11, still predominate in genital warts [21,22].

Prophylactic HPV vaccination is believed to provide protection against infection through induction of antibodies, which are present at the sites of minor epithelial trauma and bind to virus there, preventing the virus from entering the basal epithelial cells [23]. The currently licensed HPV vaccines do not assist with viral clearance, i.e. they are not therapeutic, but, in already or previously infected females, they may prevent new infection with other HPV types, reinfection, and further spread of virus to other areas of the genital tract. This has been suggested based on observations of lower rates of recurrent disease in vaccinated females previously treated for high grade cervical disease [24]. These principles also apply to the vaccination of already sexually active females who are immunocompromised, as they may benefit from vaccination in these ways even if they have already been exposed to HPV [9]. Ideally, HPV vaccination should have occurred routinely in early adolescence prior to sexual activity but immunocompromised females have a greater potential capacity to benefit from later vaccination than the general population. The nonavalent HPV vaccine provides protection against the low risk types HPV 6 and 11 as well as seven oncogenic HPV types (16,18,31,33,45,52,58) and might be considered even in those who have already received either quadrivalent or bivalent HPV vaccine to extend the number of HPV types protected against. For females who previously commenced but did not complete HPV vaccination courses prior to onset of immunomodulatory therapy, the course can be completed with nonavalent HPV vaccine without needing to restart the course.

The risk of long term exposure to immunomodulatory therapies may be considerable as treatment tends to be initiated at a young age and continue for decades, or even lifelong. Existing studies are limited by sample size, and to date, azathioprine is the only immunosuppressant commonly used in autoimmune conditions shown to increase the risk of cervical cancer (HR of 1.4 [95% CI: 0.9–2.1]) [25]. The risk of cervical cancer was twofold in females who received a high cumulative dose of >300 defined daily doses of azathioprine (HR of 2.2, 95% CI 5 1.2–3.9). Despite the increasingly specific mode of action of newer immunotherapies, many have only been available for short periods and data on long-term effects such as HPV infection and/or cervical cancer are unknown. Moreover, the risk attributable to various therapies is difficult to separate from factors such as medication dose, duration, concurrent medications and underlying disease severity. Given that oncogenesis from HPV infection typically develops over decades, ongoing surveillance and study is warranted [26] and a pro-active approach to safety is important.

Several strengths of this study should be noted. We examined a large cohort of Australian females and include a wide spectrum of rare auto-immune diseases and HIV. The inclusion of an HIV cohort, where the high risk of cervical dysplasia and cancer is well established and associated with the degree of immunodeficiency, provides a unique context to these results. This is the first study to report rates of cervical dysplasia or cervical cancer in patients with psoriatic arthritis or enteropathic arthritis. Age is known to be a major confounder of analysing cervical dysplasia data and we therefore report the rates and risks with an entry-age-adjusted age-scale. The distribution of normal and abnormal screening results at baseline in the cohort is similar to that for all Victorian females as reported by registry data (cohort vs registry: 91.1% vs 91.1% negative, 5.5% vs 4.6% low grade, 1.5 vs1.3% high grade) [13]. Contemporary guidelines recommended that immunocompromised patients underwent yearly Pap tests, which increased the chance of identifying transient infections. Although our analysis adjusted for frequency of testing, it cannot fully remove this increased ascertainment bias of cervical abnormalities in our analyses.

Our data did not include duration of disease, presence and duration of immunomodulatory therapy, HPV vaccination status (which is known to reduce cervical dysplasia risk in young females in Victoria) [27] or risk factors for cervical cancer such as cigarette smoking, use of oral contraception, multiparity, body mass index, sexual history, and young age at first full-term pregnancy [28]. We did control for age, number of tests, socioeconomic status and remoteness of residence, which provides some control for underlying demographic characteristics which may be correlated with some of these factors (e.g. smoking). As such, although this study provides insights as to comparative risks within this cohort, it cannot be used to estimate risk at the level of the individual. Whilst we identified a higher proportion of Aboriginal women within the autoimmune than control cohort, caution is warranted in interpretation given potential limitations of self-identification in emergency department data, the method used to classify disease status, and limited corroborating evidence of an increased risk of these diseases in Aboriginal women [29,30]. Variables such as HPV vaccination status, cigarette smoking, use of oral contraception, multiparity, body mass index, sexual history, and young age at first full-term pregnancy would be important to consider in future research regarding risk of cervical dysplasia. Data linkage at a national level, and with clinical databases which include variables such as type, dose and duration of immunomodulatory therapies, would increase power and provide a more accurate model of risk of cervical abnormalities in populations with these rare autoimmune conditions.

The study design included females who had ever presented to public hospital emergency departments in Victoria over a period of 14 years. However, as the majority of patients with autoimmune conditions are well and managed in outpatient settings, there may be an over-representation of females with more active disease included in the autoimmune cohort. About 20% of Australians with chronic illnesses attend emergency departments every year; this study therefore would capture many, but not all, females living with autoimmune conditions or HIV in our community [31]. Further, a primary and two additional diagnoses are recorded per patient per emergency department attendance. This may lead to under-ascertainment of females with autoimmune conditions or HIV, as these conditions may not be recorded if not directly relevant to the hospital episode or if other diagnoses are recorded as a priority.

## Conclusions

In summary, most autoimmune conditions appear to increase the risk of cervical HPV infection and related disease. These data have important public health implications especially in countries where cervical screening programs are lacking. The expansion of eligibility for funded vaccination to females with these conditions to optimise cervical cancer prevention programs should be considered. Further research to better understand risks relating to duration and type of immune-therapy and other known risk factors are needed in females with auto-immune conditions.

## Supporting information

S1 Appendix ICD-10-AM codes for multiple sclerosis, HIV and other autoimmune conditions. (DOCX)

S2 Appendix Conditions of interest. (DOCX)

S3 Appendix Covariates. (DOCX)
