# Supplementary material for: Increased risk of cervical dysplasia in females with autoimmune conditions—Results from an Australia database linkage study
Source: PLoS One. 2020 Jun 18;15(6):e0234813. doi: 10.1371/journal.pone.0234813 (PMC7302686; doi:10.1371/journal.pone.0234813)
Supplement: S1 Appendix — (DOCX) [file pone.0234813.s001.docx]

The following ICD-10-AM codes were applied to the Victorian Emergency Minimum Dataset (VEMD) to determine if an female was diagnosed with Multiple Sclerosis, HIV, or other autoimmune conditions:

**Human Immunodeficiency Virus (HIV)**

B20 Human immunodeficiency virus [HIV] disease resulting in infectious and parasitic diseases

B21 Human immunodeficiency virus [HIV] disease resulting in malignant neoplasms

B22 Human immunodeficiency virus [HIV] disease resulting in other specified diseases

B23.x Human immunodeficiency virus [HIV] disease resulting in other conditions

B24 Unspecified human immunodeficiency virus [HIV] disease

**Functional disorders of polymorphonuclear neutrophils**

D71 Functional disorders of polymorphonuclear neutrophils

**Immunodeficiency**

D80.x Immunodeficiency with predominantly antibody defects

D81.x Combined immunodeficiencies

D82.x Immunodeficiency associated with other major defects

D83.x Common variable immunodeficiency

D84.x Other immunodeficiencies

**Multiple Sclerosis**

G35 Multiple sclerosis

**Other acute disseminated demyelination**

G36.0 Neuromyelitis optica (Devic)

G36.9 Acute disseminated demyelination, unspecified

**Crohn's Disease (Regional Enteritis)**

K50.x Crohn's disease (regional enteritis)

**Ulcerative Colitis**

K51.x Ulcerative colitis

**Rheumatoid Arthritis (RA)**

***Seropositive Rheumatoid Arthritis***

M05.xx Seropositive rheumatoid arthritis

***Other Rheumatoid Arthritis***

M06.0x Seronegative rheumatoid arthritis

M06.2x Rheumatoid bursitis

M06.3x Rheumatoid nodule

M06.8x Other specified rheumatoid arthritis

M06.9x Rheumatoid arthritis, unspecified

**Psoriatic and Enteropathic Arthropathies**

***Psoriatic Arthropathies (PsA)***

L40.5 Arthropathic psoriasis (M07.0−* − M07.3−*, M09.0−*)

M07.0x Distal interphalangeal psoriatic arthropathy (L40.5)

M07.1x Arthritis mutilans (L40.5)

M07.2x Psoriatic spondylitis (L40.5)

M07.3x Other psoriatic arthropathies (L40.5)

M09.0x Juvenile arthritis in psoriasis (L40.5)

***Enteropathic Arthropathies (EA)***

M07.4x Arthropathy in Crohn's disease (regional enteritis) (K50.−)

M07.5x Arthropathy in ulcerative colitis (K51.−)

M07.6x Other enteropathic arthropathies

M09.1x Juvenile arthritis in Crohn's disease (regional enteritis) (K50.−)

M09.2x Juvenile arthritis in ulcerative colitis (K51.−)

**Systemic Lupus Erythematosus**

M32.x Systemic lupus erythematosus

**Systemic Sclerosis**

M34.x Systemic sclerosis

**Ankylosing Spondylitis (AS)**

M08.1x Juvenile ankylosing spondylitis

M45.xx Ankylosing spondylitis

**Systemic Nectrotizing Vasculitides**

M30.0 Polyarteritis nodosa

M30.1 Polyarteritis with lung involvement (Churg−Strauss)

M30.2 Juvenile polyarteritis

M30.8 Other conditions related to polyarteritis nodosa

M31.0 Hypersensitivity angiitis

M31.1 Thrombotic microangiopathy

M31.3 Wegener's granulomatosis

M31.4 Aortic arch syndrome (Takayasu)

M31.5 Giant cell arteritis with polymyalgia rheumatica

M31.6 Other giant cell arteritis

M31.7 Microscopic polyangiitis

M31.9 Necrotising vasculopathy, unspecified

**Dermatomyositis & Polymyositis (DMPM)**

M33.0 Juvenile dermatomyositis

M33.1 Other dermatomyositis

M33.2 Polymyositis

M33.9 Dermatopolymyositis, unspecified

M36.0 Dermato(poly)myositis in neoplastic disease (C00−D48)

**Sjogren’s Syndrome (SjS)**

M35.0 Sicca syndrome (Sjogren)

**Mixed Connective Tissue Disease (MCTD)**

M35.1 Other overlap syndromes

M35.8 Other specified systemic involvement of connective tissue

M35.9 Systemic involvement of connective tissue, unspecified

x – A number ranging from 0 to 9
