# Supplementary material for: Increased risk of cervical dysplasia in females with autoimmune conditions—Results from an Australia database linkage study
Source: PLoS One. 2020 Jun 18;15(6):e0234813. doi: 10.1371/journal.pone.0234813 (PMC7302686; doi:10.1371/journal.pone.0234813)
Supplement: S2 Appendix — (DOCX) [file pone.0234813.s002.docx]

The following conditions of interest were categorised in the following way:

- Arthropathies: Psoriatic and Enteropathic Arthropathies, Ankylosing spondylitis
- HIV: Human Immunodeficiency Virus, Immunodeficiency
- IBD: Crohn’s Disease, Ulcerative Colitis
- MS: Multiple Sclerosis, Acute disseminated demyelination
- RA: Rheumatoid Arthritis
- SLE/MCTD: Systemic Lupus Erythematosus & Systemic Sclerosis; Sjogren’s Syndrome, Mixed Connective Tissue Disease, Dermatomyositis & Polymyositis

*Note:* No cases of the following conditions were identified in the analysis cohort:

- Immunodeficiency
- Functional disorders of polymorphonuclear neutrophils
- Other acute disseminated demyelination
- Systemic Sclerosis
- Ankylosing Spondylitis
- Systemic Necrotizing Vasculitides
- Dermatomyositis & Polymyositis
- Sjogren’s Syndrome
- Functional disorders of polymorphonuclear neutrophils
- Systemic Necrotizing Vasculitides
