# Supplementary material for: Increased risk of cervical dysplasia in females with autoimmune conditions—Results from an Australia database linkage study
Source: PLoS One. 2020 Jun 18;15(6):e0234813. doi: 10.1371/journal.pone.0234813 (PMC7302686; doi:10.1371/journal.pone.0234813)
Supplement: S3 Appendix — (DOCX) [file pone.0234813.s003.docx]

**Age at Study Entry**

Age at study entry was calculated by subtracting the date of birth of each female from the date of study entry. However, only the month and year of date of birth was provided in the VCCR dataset. Therefore, to calculate the age at study entry, we assumed each females date of birth was on the 15th day of each month.

**Socio-economic Status**

Socio-economic status was derived by using the standard Australian area based measure assigned through postcode of residence (ABS SEIFA^#^). Each socio-economic status classification is listed below:

- Lowest (Decile 1-2)
- Low-Med
- Medium
- Med-High
- Highest (Decile 9-10)

*^#^Australian Bureau of Statistics, 2033.0.55.001 - Census of Population and Housing: Socio-Economic Indexes for Areas (SEIFA), Australia, 2016.*

**Remoteness Area**

Area of remoteness was derived by using the standard Australian area based measure assigned through postcode of residence (ABS ASGS*). Each remoteness area classification is listed below:

- Major City
- Inner Regional
- Outer Regional
- Remote/Very Remote

**Australian Bureau of Statistics, 1270.0.55.005 - Australian Statistical Geography Standard (ASGS): Volume 5 - Remoteness Structure, Australia, 2016.*

**Aboriginal and/or Torres Strait Islander Status**

To derive a woman’s Aboriginal and/or Torres Strait Islander status from the VEMD, the primary method implemented was the following:

Ever Aboriginal and/or Torres Strait Islander - An female is recorded as Aboriginal and/or Torres Strait Islander if at least one record (in a dataset) or one dataset within the study period identifies them as Aboriginal and/or Torres Strait Islander (AIHW 2012^$^).

*^$^Australian Institute of Health and Welfare and Australian Bureau of Statistics 2012. National best practice guidelines for data linkage activities relating to Aboriginal and Torres Strait Islander people.*

**Country of Birth (Major Regions)**

The major grouping classification for each female’s country of birth was determined from the Standard Australian Classification of Countries (ABS SACC^%^). This document is derived by the ABS and is freely available on their website. Each major region classification is listed below:

- Americas
- North Africa & The Middle East
- North-East Asia
- North-West Europe
- Oceania & Antarctica
- South-East Asia
- Southern & Central Asia
- Southern & Eastern Europe
- Sub-Saharan Africa
- Not Classified

*^%^Australian Bureau of Statistics, 1269.0 - Standard Australian Classification of Countries (SACC), Second Edition, Australia, 2016.*
